# Validation method for determining enrofloxacin and tylosin levels in broiler liver, kidney, and muscle using high-performance liquid chromatography

**DOI:** 10.14202/vetworld.2022.268-274

**Published:** 2022-02-08

**Authors:** Agustina Dwi Wijayanti, Rahmad Dwi Ardiansyah, Anggi Muhtar Pratama, Aris Haryanto, Ida Fitriana

**Affiliations:** 1 Department of Pharmacology, Faculty of Veterinary Medicine, Universitas Gadjah Mada, Jl. Fauna no. 2 Karangmalang, Yogyakarta, Indonesia; 2 Department of Biochemistry, Faculty of Veterinary Medicine, Universitas Gadjah Mada, Jl. Fauna no. 2 Karangmalang, Yogyakarta, Indonesia

**Keywords:** enrofloxacin-tylosin combination, high-performance liquid chromatography, validation method

## Abstract

**Background and Aim:**

Enrofloxacin and tylosin can be combined into an antibiotic formulation which is expected to have a broader range of antibacterial activity against various infections in broilers. Validation method analysis of the levels of these two active compounds needs to be done for future use in pharmacokinetic or residual studies. The present study aims to determine a suitable validation method of isocratic high-performance liquid chromatography (HPLC) to measure the concentration of antibiotic combinations in the broiler liver, kidney, and muscles.

**Materials and Methods:**

The combination of enrofloxacin and tylosin in the liver, kidney, and muscle was validated by HPLC method to find the procedures, processes, equipment, and systems used, consistently provides the appropriate results. The chromatography system consisted of an Octadecyl-silica column of 5 mm in diameter and 150 mm in length with a mobile phase of a mixture of 0.05 M monobasic sodium phosphate (pH 2.5) and acetonitrile (65:35 v/v). The solution was detected at a wavelength of 280 nm, 30°C, a flow rate of 1 mL/min, and an injection volume of 20 mL. The combination antibiotics powder was produced from PT Tekad Mandiri Citra, Bandung, Indonesia, and broiler tissues obtained from day-old chick broilers maintained for 30 days with free antibiotic feed.

**Results:**

Validation of a combination solution of enrofloxacin and tylosin shows the linearity values of enrofloxacin and tylosin in the liver, kidney, and muscles as r^2^=0.9988, r^2^=0.9999, r^2^=0.9997, r^2^=0.9989, r^2^=0.9978, and r^2^=0.9962. The accuracy and precision values of enrofloxacin in the liver, kidney, and muscles were 5.53, 6.23, and 6.93, respectively. The values of accuracy and precision of tylosin in the liver, kidney, and muscles were 10.43, 4.63, and 7.16%, respectively. The retention times for enrofloxacin and tylosin were 1.945-2.000 min and 4.175-4.342 min. The limit of detection (LOD) and limit of quantity (LOQ) values for enrofloxacin were 3.03 and 10.1 μg/g, respectively. In contrast, the LOD and LOQ values for tylosin were 9.05 and 30.17 μg/g, respectively.

**Conclusion:**

The value of linearity, accuracy, precision, specificity, and sensitivity of the combined solution of enrofloxacin and tylosin showed promising results.

## Introduction

Broiler farms are generally susceptible to viral, bacterial, fungal, and parasitic infections which might increase due to poor quality environmental conditions [1,2]. Suitable antibiotic therapies are used to treat infections caused by Gram-positive and Gram-negative bacteria [3-5]. Enrofloxacin is a potent antibiotic having bactericidal action by affecting bacterial DNA topoisomerase [6-8]. Several bacteria such as *Mycoplasma gallisepticum* and *Mycoplasma synoviae* have been reported to be resistant to enrofloxacin [9-11]. However, a combination of antibiotics can be used as an alternative to avoid antibiotic resistance. Tylosin is an antibiotic of macrolide group having bacteriostatic action that can bind to 23S rRNA of the 50S ribosomal subunit, thereby inhibiting protein synthesis. Moreover, they induce the dissociation of peptidyl-tRNA from the ribosome during translocation [12-14]. The combination of enrofloxacin and tylosin is expected to produce better effectiveness, especially in poultry therapy. Furthermore, this combination has been previously studied to check for any damaging effects on the liver and kidney. However, it is reported to be safe when administered at therapeutic doses [15].

Pharmacokinetic simulation can explain the retention of drugs in the body until excretion and can be used to determine residual levels per time unit. One of the analytical methods used for pharmacokinetic and residual studies is high-performance liquid chromatography (HPLC) [16]. Single-drug formulations in the body are usually determined by one HPLC validation method. HPLC validation methods need to be developed to measure the concentration of two or more compounds in broiler tissue [17]. Validation of analytical methods is a process of the procedures, systems, processes, materials, and tools so that if used, it would consistently provide the expected results. Validation of analytical methods is an act of parameter assessment to obtain a standard in the test of an ingredient. Some of the analysis parameters are accuracy, precision, selectivity, linearity, the limit of detection (LOD), and quantity (LOQ).

This study aimed to validate the HPLC analysis method for determining concentrations of a combination of enrofloxacin and tylosin *in vitro* in the liver, kidney, and muscles of the broiler using a validated analytical method.

## Materials and Methods

### Ethical approval

The study was approved by Ethics Commission of the Faculty of Veterinary Medicine, Gadjah Mada University (Approval number 0042/EC-FKH/Int/2019).

### Study period, area, and procedure

The study was conducted from September 2019 until February 2020 in Pharmacology Laboratory and study center of animal, Faculty of Veterinary Medicine Gadjah Mada University. Methods include treating animals and tissues sampling, preparing the standard curve of tylosin-enrofloxacin formulation in the mobile phase and drug spiking the liver, kidney, and muscles, and determining the value of the analyzed parameters. The combination product consisted of 125 mgtylosin tartrate and 125 mgenrofloxacin HCl in 1000 mg powder of formulation and was obtained from PT Tekad Mandiri Citra, Bandung, Indonesia. Analysis of the concentration of tylosin-enrofloxacin as serial concentration from stock solution and in the liver, kidneys, and muscle samples was carried out using theHPLC tool (Shimadzu version 6.1, Tokyo, Japan) based on the Animal Drug Residue Analysis method [5,18-20].

### Animal handling and sampling

Three broilers were kept in postal pens of experimental animals starting from day-old chicken and given antibiotic-free BR-1 feed and *ad libitum* drinking water. After30 days of age, broilers weighing 1-1.5 kg were slaughtered by halal slaughter method, then dissected. The liver, kidneys, and muscles were collected and stored in a freezer until analysis on the HPLC equipment. The broiler tissues used in spiking analysis measure the drug concentration from blank sample tissues.

### The mobile phase construction and HPLC preparation

The HPLC tool was set at a temperature of 30°C, a detector at a wavelength of 280 nm, and a flow rate of 1 mL/min. The mobile phase used is a mixture of 0.05 M monobasic sodium phosphate (pH 2.5) and acetonitrile in the ratio of 65:35 v/v. The mobile phase mixture was adjusted to pH 2.5 by adding a few drops oftriethylamine (Merck KGaA, Darmstadt, Germany).

### Tissue sample extracts production

Analysis of the concentration of enrofloxacin-tylosin in the liver, kidney, and muscle samples was by first extracting tissue using a modified method of Widiastuti [20]. The liver, kidney, and muscles were chopped and crushed until smooth; each was weighed as much as 1 g and put in acentrifuge tube (Corning^®^, Merck, Germany) then 2.5 mL of 1% acetonitrile acid (1 mL of acetic acid anhydrous in 100 mL acetonitrile; J.T. Baker, Solusorb, USA) were added. The samples and chemicals were then vortexed for 5 min and centrifuged at 3000× *g* for 10 min. The supernatant was separated and kept in a water bath for 10 min. After drying, 1.5 mL phosphate buffer (pH 7.4) and 2 mL N-hexane were added; it was mixed with vortex for 5 min and centrifuged at 3000× *g* for 10 min. Then, the supernatant was taken, hexane was removed, and the procedure was repeated thrice. The collective supernatant obtained was then centrifuged at 2500× *g* for 15 min, then filtered, and injected into HPLC as much as 20 μL. The concentration of enrofloxacin-tylosin in the liver, kidney, and muscles was estimated to create a standard curve of the drug in the tissue.

### Building enrofloxacin-tylosin standard curves in the liver, kidney, and muscles

The standard curve of the combined solution was first prepared by injecting serial concentrations diluting of 10, 50, and 100 μg/mL from stock solution 1 mg/mL into the HPLC system. The standard curves of the drug in the liver, kidney, and muscle samples were then made by spiking the drug in the sample blank. The results of the extraction of a blank sample of liver, kidney, and muscle were combined with a standard solution of enrofloxacin-tylosin with the concentrations of 10, 50, and 100 μg/g, respectively. To obtain a linear line equation, each concentration was injected as much as 20 μL with three repetitions. The standard curve was created for the peak area of each drug concentration.

### Determination of the value of accuracy, precision, specificity, and sensitivity of the HPLC method

The accuracy and precision values of the analysis method were used to prepare the standard curve of enrofloxacin in the liver, kidneys, and muscles using the method above. The accuracy value (% recovery) is calculated by dividing the measured concentration by the actual concentration multiplied by 100%. The precision value is expressed by the coefficient of variation (CV%), which is calculated to compare standard deviations with the average measured concentration multiplied by 100% [21].

Specificity was determined by observing at the peak area profile and retention time on the chromatogram of the standard solution of enrofloxacin-tylosin (concentration 10, 50, and 100 μg/g) and comparing the chromatogram blank and spiking the standard solution of a tylosin-enrofloxacin concentration of 10 μg/g in the liver, kidney, and muscles.

The sensitivity of the HPLC is determined by setting LOQ and LOD values. The limit of quantification is the smallest number of analytes in a sample that still meets the particular criteria. At the same time, the LOD is the smallest number of analytes in a sample that can still be detected even though it could not always be quantified and gives a significant response compared to the blank [21].

## Results

**Figure-1:**
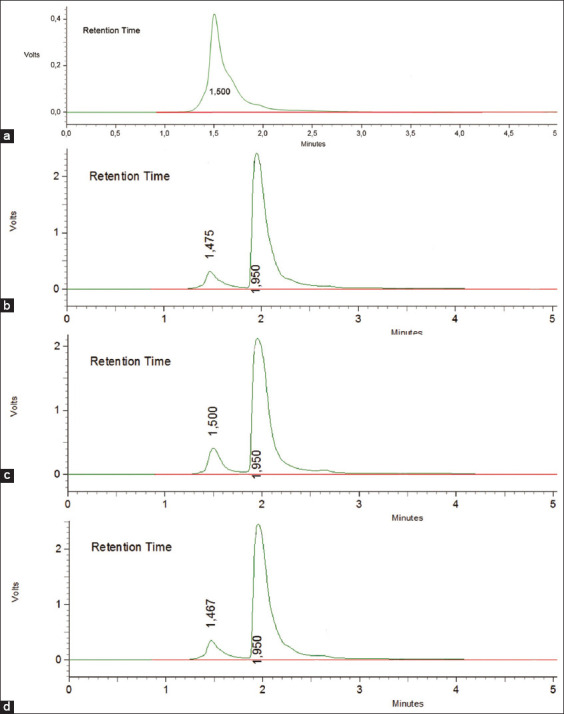
Standard enrofloxacin chromatogram. (a) Chromatogram blank (without drugs) muscle; (b) enrofloxacin spiking chromatogram concentration of 10 μg/g in the liver (retention time 1.950 min); (c) enrofloxacin spiking chromatogram concentration of 10 μg/g in the kidney (retention time 1.950 min); (d) enrofloxacin spiking chromatogram concentration of 10 μg/g in muscle (retention time 1.950 min).

**Figure-2:**
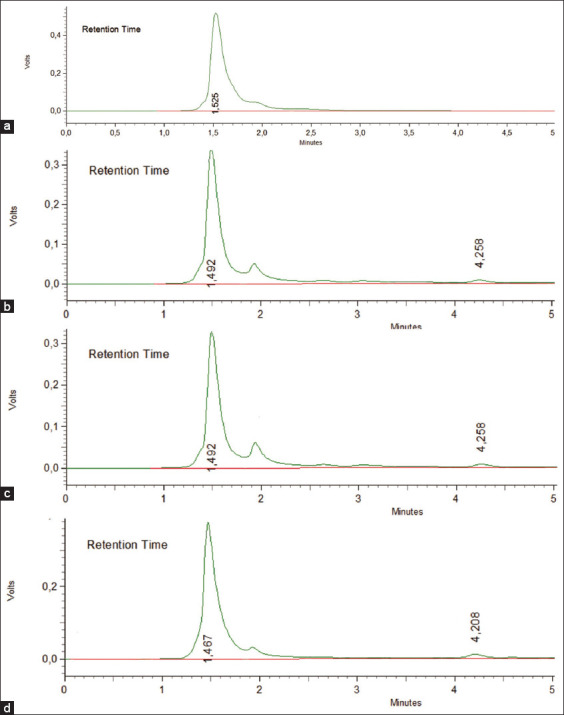
Standard tylosin chromatogram. (a) Chromatogram blank (without drugs) muscle; (b) tylosin spiking chromatogram concentration of 10 μg/g in the liver (retention time 4.258 min); (c) tylosin spiking chromatogram concentration of 10 μg/g in the kidney (retention time 4.258 min); (d) tylosin spiking chromatogram concentration of 10 μg/g in muscle (retention time 4.208 min).

**Figure-3:**
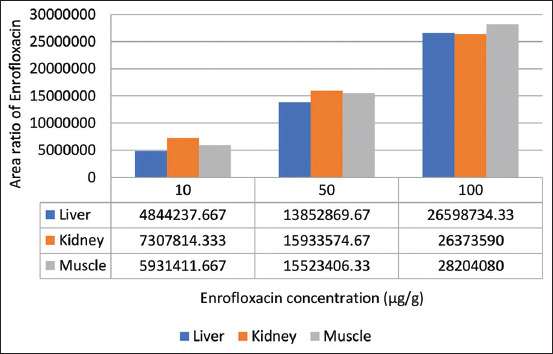
Enrofloxacin calibration graph for liver, kidney, and muscle with the concentration of 10 μg/g, 50 μg/g, and 100 μg/g.

**Figure-4:**
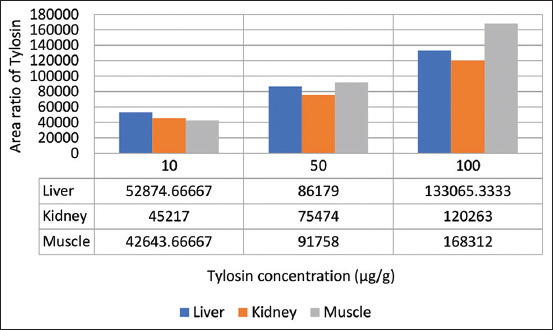
Tylosin calibration graph in liver, kidneys, and muscles with the concentration of 10 μg/g, 50 μg/g, and 100 μg/g.

The validation of the method of determining the levels of enrofloxacin and tylosin in the liver, kidneys, and muscles using HPLC was performed to see linearity, accuracy, precision, specificity, and sensitivity [22-24]. Before drug spiking in tissues, the standard stock solution of drug formulation was carried out to find the linearity of three different concentrations (10, 50, and 100 μg/g). The chromatogram showed separated peaks of retention times of enrofloxacin and tylosin as 1.945-2.000 and 4.175-4.342 min, respectively (Figures-1 and 2). The standard equations of the enrofloxacin and tylosin curves in the liver, kidneys, and muscles are Y=bx+a, with R^2^, the coefficient of determination, and r, the coefficient of correlation. Equations of enrofloxacin curve were=42,258x+2,178,206 and R^2^ 0.9988 (liver), Y=211,717x+5,246,737 and R^2^ 0.9999 (kidney), and Y=247,726x+3,340,928 and R^2^ 0.9997 (muscle), respectively, while the equations of tylosin curves were Y=1401.8x+26,140 and R^2^ 0.9989 (liver), Y=836.38x+35,711 and R^2^ 0.9978 (kidney), Y=1401.8x+26,140 and R^2^ 0.9962 (muscle), respectively. The linearity graph of the combination of enrofloxacin and tylosin is shown in Figures-3 and 4.

The validation results showed that the accuracy value of enrofloxacin in the liver was 10 μg/g at 11.62%, 50 μg/g at 4.07%, and 100 μg/g at 1.81%. The validation results showed that the accuracy value of enrofloxacin in the kidney was 10 μg/g at 8.32%, 50 μg/g at 6.49%, and 100 μg/g at 3.71% and in muscle was 10 μg/g at 14.29%, 50 μg/g at 3.90%, and 100 μg/g at 3.18%, respectively.

The validation results showed that the accuracy of tylosin in the liver was 10 μg/g at 11.02%, 50 μg/g at 10.19%, and 100 μg/g at 10.78%, in the kidney was 10 μg/g at 2.57%, 50 μg/g at 4.00%, and 100 μg/g at 7.51%, and in the muscle was 10 μg/g at 12.21%, 50 μg/g at 9.01%, and 100 μg/g at 1.49%. In addition to accuracy, the recovery value (% recovery) is also calculated by comparing the measured concentration of enrofloxacin or tylosin with the actual level of enrofloxacin or tylosin multiplied by 100%. The values of enrofloxacin recovery in the liver, kidney, and muscle were 92.39-125.88, 85.86-109.31, and 94.47-124.77%, respectively. The values of tylosin recovery in the liver, kidney, and muscle were 82.17-124.86, 90.50-117.24, and 86.48-113.53%, respectively.

The precision (CV%) values of enrofloxacin in the liver, kidneys, and muscles were 5.53, 6.23, and 6.93%, respectively. Precision values of tylosin in the liver, kidney, and muscles were 10.43, 4.63, and 7.16%, respectively.

The results show that the specificity of enrofloxacin with the time of retention in the liver, kidney, and muscles was1.950-2.000, 1.950-1.958, and 1.945-1.950 min, respectively. The specificity of tylosin with the time lease in the liver, kidney, and muscles, respectively, was as follows: 4.258-4.342, 4.175-4.342, and 4.208-4.242 min. The retention time and chromatogram peak between the two compounds were well separated.

The LOQ values of enrofloxacin in the liver, kidney, and muscles were 22.2, 5.85, and 10.1 μg/g, respectively. The LOQ values of tylosin in the liver, kidney, and muscle were 21.32, 30.17, and 39.17 μg/g, respectively. The LOD values of enrofloxacin in the liver, kidney, and muscles were 6.66, 1.76, and 3.03 μg/g, respectively. The tylosin LOD values in the liver, kidney, and muscles were 6.39, 9.05, and 11.75 μg/g, respectively.

## Discussion

Our results demonstrated that HPLC UV detection 280 nm can be used to determine the concentration of enrofloxacin and tylosin. As per Ghanem and Abu-Lafi [25], the optimum wavelength of 287 nm was used to detect tylosin tartrate with a column temperature of 25°C and a flow rate of 1 mL/min. Based on the research of Karademir *et al*. [26], a wavelength of 280 nm was used for the detection of enrofloxacin. The peak of analyte (enrofloxacin and tylosin) was easy to identify from the impurities peak of other unknown substances in the feed matrix.

Linearity is indicated by the value of r (correlation coefficient). The r values of enrofloxacin in the liver, kidneys, and muscles were 0.9994, 0.9999, and 0.9999, respectively, while the r values of tylosin in the liver, kidney, and muscles were 0.9999, 0.9989, and 0.9981, respectively. Linear regression data showed good linearity for all the analytes with correlation coefficient (r) in the range of 0.998-0.999 [24]. The results of this study indicate an excellent linearity value (r>0.9999) as in the study by Anacleto *et al*. [27].

The accuracy of the sample combination of enrofloxacin and tylosin was made in three standards with known concentrations of 10, 50, and 100 μg/g. The validation results showed that the accuracy values of enrofloxacin and tylosin levels in the liver, kidney, and muscles were good. Based on the research of Yaneva *et al*. [28], the detection of tylosin using HPLC with a mobile phase composed of acetonitrile and 0.1 M H3PO4 (60:40, v/v), flow rate to 0.8 mL/min, temperature 30°C, and wavelength 290 nm showed an accuracy value of 0.89%. An accuracy value of <10% shows promising results for analyte testing. A study by Ancleto *et al*. [27] showed that enrofloxacin detection using HPLC with a mobile phase water and acetonitrile (87:13, v/v) plus 0.1% trifluoroacetic acid, flow rate to 3 mL/min, and wavelength detection of 280 nm fetched an accuracy value of 0.45-1.1%, the accuracy is good as the value is less than 10%. According to Hakim [29], the accuracy of using the sample extraction method should be no less than 75% or a systematic value of no more than 25%. Meanwhile, the accuracy received was ±15%, except for the LOQ value of ≤20%. The accuracy and precision test results did not exceed ±15% [30].

Good precision will produce repeated measurements at the same level with relatively low variation. The precision (CV%) values of enrofloxacin and tylosin in the liver, kidney, and muscles were good. The results of this study are in accordance with Chakravarthy *et al*. [31], with a CV% value of 1.9% for the detection of enrofloxacin using HPLC. Tylosin tested by HPLC using a mobile was an isocratic combination of water: acetonitrile (70:30; v/v) adjusted to pH of 5.5 using 0.1 N acetic acid, flow rate 1 mL/min, wavelength 287 nm, and column temperature 25°C shows CV values % of 1.16%, the value shows good results [24]. According to Lindholm [32], an analytical method for biological sample research was said to meet requirements if the CV% was ≤15%. The results of this study indicate that the CV% is in accordance with the literature.

Specificity is the ability of an analytical method to carefully measure compounds that are measured without interference from other compounds. The specificity of the analytical method in this study was determined by looking at the peak area profile on a blank chromatogram and spiking a standard solution of enrofloxacin and tylosin in the liver, kidney, and muscles. Chromatograms containing standard peak area profiles of enrofloxacin and tylosin at concentrations of 10, 50, and 100 mg/g are shown in Figures-1 and 2.

The sensitivity of the test method in this study was determined by identifying the LOQ and LOD values. The LOQ is the smallest quantity of compounds in a sample that still meets the meticulous and criteria. At the same time, the LOD is the smallest amount of compound in a sample that can still be detected even though it cannot always be quantified and gives a significant response compared to the blank [32,33]. Based on research by Ghanem and Abu-Lafi [25], the values of LOD and LOQ tylosin are 5.6 and 18.7 μg/mL. Results of Moudgil *et al*. [23] detected enrofloxacin using HPLC with a mobile phase of methanol: acetonitrile (75:25, v/v) with a gradient elution of 90:10 aqueous phase, a flow rate to 0.8 mL/min, and a column temperature of 27°C showing LOD values and LOQ of 18.9 and 57.3 ng/mL. Based on research by Anacleto *et al*. [27], the LOD and LOQ values of enrofloxacin were 2.24 and 7.46 μg/mL. The LOD and LOQ values in this study are not much different from the previous literature, which shows that the method in this study can be used to detect enrofloxacin and tylosin. According to the European Commission, maximum residue limits of enrofloxacin and tylosin are range from 100 to 300 μg/kg in broilers tissues [1,2]. The current study needs improvement for measuring LOQ and LOD values, as there are other limitations in measuring the residual value of these two drug compounds.

## Conclusion

Optimum conditions for analysis of enrofloxacin and tylosin in the liver, kidneys, and muscles *in vitro* as a standard in HPLC using Octadecyl-silica columns are 5 mm in diameter and 150 mm length; mobile phase mixture of 0.05 M monobasic sodium phosphate (pH 2.5) and acetonitrile in the ratio of 65:35 v/v; a temperature of 30°C; wavelength of 280 nm; and a flow rate of 1 mL/min. The validation results show that our bioanalysis method has fulfilled the criteria of linearity, accuracy, precision, specificity, and sensitivity in accordance with the provisions contained in the European Medicines Agency Guideline, and hence, it could be used for the analysis of a combination of enrofloxacin and tylosin in the liver, kidney, and muscles of broiler.

## Authors’ Contributions

ADW, IF, and AH: Designed the study. RDA, AMP, and IF: Field research and sample analysis in the laboratory. All authors wrote, edited, read, and approved the final manuscript.
